# Chemical Biology is.....

**DOI:** 10.1186/1752-153X-1-5

**Published:** 2007-02-19

**Authors:** Elizabeth L Ostler

**Affiliations:** 1 School of Pharmacy and Biomolecular Sciences, University of Brighton, Cockcroft Building, Moulsecoomb, Brighton BN2 4GJ. UK.

## Abstract

Chemical Biology is a relatively new field, and as such is not yet simply or succinctly defined. It includes such a wide range of fundamental problems that this commentary could only include just a few snapshots of potential areas of interest. Overarching themes and selected recent successes and ideas in chemical biology are described to illustrate broadly the scope of the field, but should not be taken as exhaustive. The Chemical Biology Section of Chemistry Central Journal is pleased to receive manuscripts describing research into all and any aspects of the subject.

## Background

One always expects a commentary of this sort to begin with a clear and succinct definition of the scope and boundaries of the topic under discussion. Unfortunately, when asked to define Chemical Biology, most researchers seem to be lost for an apt description. In fact, a brief survey of colleagues led to a consensus of "Chemical Biology, like good taste, is somewhat hard to pin down, but you know it when you see it".

Perhaps some of the problem lies in the sheer range of topics that fall under the umbrella of the discipline. A small sample of the literature being produced by Chemical Biology institutes and departments around the world included articles ranging from enzymology to medicinal chemistry, and from structural biology to single cell proteomics. The list of subject groups and departments collaborating on Chemical Biology projects is equally varied, reflecting a tremendous diversity of specialisations contributing to the field.

Some attempts at definition have focused on what Chemical Biology is not. It is not a service for biologists with chemists acting as an upgraded version of the Sigma catalogue. (One biologist who clearly disagreed with this view once asked me "how many new compounds can you make in a week?") Equally it is not about a remora-like relationship where one scavenges ever higher impact factor articles by adding some biological data to what was a rather lightweight synthetic paper. This field should represent the best and brightest and not the worst and most mundane aspects of its predecessors.

Considering all this, a common underlying theme can be discerned; collaboration. Collaborative partnerships have made Chemical Biology into a subject in its own right. It would be extraordinary for a single person to be enough of a polymath to be at the forefront of both chemistry and biology, and thus it is all too easy to do great chemistry and weak biology or vice versa. However, where there is sufficient inclination for collaborators to learn each other's language and work on identifying the concepts that underpin a meaningful conversation, two specialists can work synergistically to address problems in a completely new way. It is this partnership, which allows cutting edge chemical concepts and tools to be brought to bear on previously impenetrable biological systems, that is the essence of Chemical Biology. Perhaps becoming a great chemical biologist is more about finding the right partner than finding yourself.

Whatever your view of Chemical Biology as a field, there is a great deal of evidence that chemistry is uniquely placed to increase our understanding of, and ability to intervene in, biological processes. Unlike their biological counterparts (principally antibodies and siRNA), synthetic small molecules may be cell-permeable, easily delivered at different doses with accurate quantitation and selectable timing, and their specificity can be modified simply. However, we have had to develop new synthetic strategies in order to generate sufficiently selective and active lead compounds. Equally new analytical techniques with improved detectivity, sensitivity and selectivity have been required to cope with complex biological systems. Methodological and technological advances have thus been almost as important as the new targets they have enabled us to investigate. I have therefore chosen to outline examples of interesting methodological advances and current targets separately. It is difficult to choose just a few current examples of exciting Chemical Biology, because recent achievements, like the subject matter, are many and varied. The selected examples do not therefore represent a comprehensive list, but are simply a selection of my favourites across the spectrum of the field.

## Discussion

### Methodological advances

#### Chemical genetics

Chemical genetics, [1] which relies on selecting small molecules for their ability to induce a biological phenotype or to interact with a particular gene product, is one of the best examples of a methodological development in lead generation. Forward (screening for phenotype) and reverse (screening for activity against a selected protein) chemical genetics, by combining medicinal chemistry, biological screening and combinatorial synthesis techniques, has enabled us to address previously intractable problems. Since developments in synthetic methodology tend to be incorporated into new library development, and improvements in analytical and biological methodology are applied to screening systems, chemical genetics research often encapsulates the best of both chemistry and biology.

#### Directed evolution

One of the most elegant examples of the extension of biological processes to ligand discovery is that of *in vitro *evolution. One of the reasons why antibodies have such good binding parameters is that *in vivo *they are subjected to rounds of expansion, selection and mutation which allow them to evolve with repeated exposure to the challenging agent. This process of evolution of activity by selection and mutation was first utilised *ex vivo *in phage display technology [2] and later using yeast or bacteria as vectors. This has been extended to *in vitro *techniques such as ribosome display [3], a wholly cell-free system for the directed evolution of peptides. Methods for the *in vitro *generation and evolution of aptamers (RNA and DNA sequences which bind specific targets) are also now well established [4]. Stability and cell permeability remain a problem, although such systems have been improved by incorporating synthetic residues, or by undertaking post selection modification. One particularly striking example of the integration of aptamer evolution with chemical modifications, utilised combinatorial chemistry techniques during the selection process to generate a new moiety which tightly binds the transactivational responsive (TAR) element of HIV-1 [5].

#### Design, induction and adaptation of new catalysts

Chemical approaches to biological systems have not only generated target-binding entities, but also novel catalysts. Stable analogues of transition-states are routinely used to generate antibodies which catalyse reactions of interest, and these have been applied to drug activation, drug detoxification, and synthetic chemistry [6]. Naturally occurring enzymes have also been engineered to alter their activities and specificities for use in synthetic, environmental and medical applications [7]. Conversely, wholly synthetic inorganic complexes have been used successfully as artificial mimics of enzymes such as catalase and superoxide dismutase [8,9].

#### Analytical methodology

There is a vast armament of traditional chemical analytical techniques (both classical and instrumental) which may be applied to the products of living systems. Experimental design, engineering and computer processing developments have allowed us to examine complexes and structures of ever increasing molecular weight and complexity with ever greater accuracy and resolution. Previously unobservable non-covalent interactions have been mapped, and the cross-talk between cellular (and extracellular) components investigated. New phenomena in quaternary structure and binding site topology are also being reported.

However, we do still have some really challenging analytical problems that only just beginning to be addressed. The phenotypic heterogeneity of supposedly identical individual cells has proved a confounding problem for molecular biologists. Standard biological techniques such as western blotting and microarraying require large numbers of cells, and can only produce an average result for a culture or tissue sample masking the intrinsic heterogeneity of populations of cells. Developments in miniaturisation and quantitative amplification methods are starting to generate more quantitative and qualitative data from single cells. Equally, new non-destructive methods, particularly those which can be used *in vivo*, are opening up whole new areas of research [10].

### Current targets

#### Control of cell division

Some of the most notable breakthroughs have been made in elucidating and intervening in the processes involved in cell division. There has been intense activity in this area not just because it is such a fundamental part of living systems, but also because of the potential for anti-cancer applications. One of the first, and perhaps best known, successes in this aspect of Chemical Biology is that of Monastrol [1]. Monastrol was selected as a result of a phenotype-based screen to identify compounds which disrupt the mitotic spindle during cell division. It was found to act by specifically inhibiting the activity of the kinesin Eg5, and has opened up a whole new aspect to cell cycle research and enabled completely new modes of inhibiting cell growth.

It should not be forgotten that the converse of uncontrolled cell division, cellular senescence, also represents an important biological target. With every division there is an increasing chance of cells entering cellular senescence, and never dividing again. This is not only a problem in terms of our ability to replace damaged tissues. In addition, cells which have entered senescence persist in tissue, and take on an altered, deleterious phenotype which is thought to contribute to ageing. Work which elucidates replicative control pathways thus also has a high likelihood of generating important information relating to the ageing process, an area in which I have a particular interest. The Chemical Biology of Ageing is really in its infancy, but the current demographic shift towards an aged population means that it is likely that this will represent one of the most important areas for progress in the future.

#### Cellular activity modulation

Whilst there are many modes of cellular regulation, membrane bound receptors represent an attractive target for small molecule-mediated control of cellular activity because of their accessibility. Small molecules which act as receptor ligands or inhibitors have been used successfully for neurological problems such as bladder control [11] and epilepsy [12], and for controlling smooth muscle activity [13] although work continues on improving the activity and selectivity over the drugs currently in use. Other receptor targets, such as those controlling the immune system and the induction of apoptosis have really only been exploited using antibody and peptide ligands, and successful synthetic modulators have yet to appear. Alternative possibilities for the chemical regulation of cellular processes including selective ion-channel modulators [14] and sequence specific DNA binding molecules (which promote or inhibit the transcription of specific genes), [15] are also being investigated.

Another type of approach is to study the chemical interactions between biological systems in their natural environment. Electrochemical methods have been used to characterise the effects of naturally produced algal exudates on the behaviour of other algae, [16] leading to the discovery of some interesting new ligands which affect growth rate and trace metal uptake.

#### Small biological molecule targets

Although natural products are popular as lead compounds, there has been rather less research devoted to small biological molecules as targets, and this remains a relatively undeveloped area. One such example, the group of compounds known as reactive oxygen and nitrogen species (RONS), has been successfully targeted, with a body of work devoted to antioxidants designed to prevent damage by soaking up the radical by-products of respiration. This approach has even been extended to inorganic complexes which mimic the actions of the antioxidant enzymes catalase and superoxide dismutase [8,9].

## Conclusion

As can be seen from the few examples given, research in Chemical Biology has a great deal of potential for improving our understanding of biological systems and thereby improving everyday lives. This has been reflected in the large number of new journals devoted to Chemical Biology that have been launched in the last few years. So why do I think that your research should be sent to this section of our journal?

I have two reasons for believing that this is a good place to publish. Firstly, Chemical Biology research covers such a range of fundamental problems that its true impact can only be felt in a journal with a wide and non-specialist readership. A discipline built on collaboration cannot be inward looking, however tempting that might be. As Section Editor I will welcome manuscripts covering any aspect of the subject given the broadest possible definition, as our journal is designed to have broad appeal.

The second reason relates to the advantages of online open access publishing. In my experience, there are two kinds of referee. The first considers themselves as a guardian of the literature, charged to ensure that only the finest, most earth-shattering research ever appears in the *Journal of the Lilliputian Chemical Society, Section B2 (abbreviated reports)*. The second type simply looks for good solid science with much less interest in whether the work will win a Nobel prize. So why do we see these differences in approach to reviewing essentially similar articles? I think that the former is so prevalent because of the pressures of paper publishing. A journal usually only receives a fixed income for each issue, be it 50 pages or 500 pages long, each page printed has costs attached to it, and each issue is limited in size by the printing process, so every article must contribute to the chance of generating subscriptions. This can lead to subtle, but sustained pressure on editors and referees to be exclusive, rather than inclusive. In contrast, online open access publishing frees editors and referees from these constraints, since information storage is relatively cheap and journal income is per article published. This new style of journal gives us a much greater freedom of action as an editorial board.

So, my editorial policy for the Chemical Biology section of *Chemistry Central Journal *will be simply this: all bad science will be rejected, all good science will be published, and all Nobel prize-winning science will be gratefully received. What better reason could there be for submitting your manuscript here?

If you have any queries about submitting a Chemical Biology research report, please feel free to contact me.
